# Correspondence

**Published:** 1887-04

**Authors:** 


### CORRESPONDENCE.

MESSRS. EDITORS :

The following editorial from the New York *Medical Record* of February 19th shows how highly esteemed are those who, under the name of Hahnemann, are continually dragging in the mire the fair name of Homœopathy.

Very truly yours,

GEO. H. CLARK.

GERMANTOWN, February 24th, 1887.

"A SPECIMEN OF HAHNEMANNIAN HOMŒOPATHY.

"We have read with great profit the last issue of *The Hahnemannian Monthly*, a journal devoted presumably to the propagation of Homœopathy and the homœopathic practice. The six original articles with which the issue opens are striking illustrations of how little Homœopathy there may be in the practice aforesaid. The first treats of fatty heart, and the treatment advised is mainly hygienic—'symptoms as they arise should be treated with the properly selected homœopathic remedy'—but it is admitted that the prognosis is bad, *i. e.*, the remedies are futile. The next article is devoted to showing the value of Boroglyceride in certain surgical affections. The third article is on climate and phthisis. The fourth describes how the writer gave a three-drachm vial of Atropin, ten drachms [?], to a patient with singultus, until the throat was dry and the pupils dilated. He next gave one-fifth of a grain of Pilocarpin, then Lachesis, then Nux vomica, then Ignatia, then Hyoscyamus, and then Gelseminum, in one minim doses. At the end of three months the patient was well, despite the polytherapy. The last communication is upon Belladonna, Agaricus, and Borax, and reads a good deal like the real jargon of dogmatic therapeutics.

"The perusal of the articles above referred to leads one to ask how much of the real Hahnemannism there is among the readers of our valuable contemporary. There are certainly more climate, Pilocarpin, and strong nervines and narcotics than anything else in the last issue."

---

January 31st, 1887.

DEAR MR. EDITOR :

Permit me to point out a mistake occurring in January's issue of your much valued journal, where there occurs some little confusion between my father and myself, at page thirty-five. My father's present address is Darlinghurst, Sydney, Australia, and *mine* is 41 Catharine Street, Liverpool. In every other respect your kind remarks of my father are perfectly correct.

Apologizing for troubling you,

Most fraternally,

FOURNESS SIMMONS.

